# Tuberculosis as a Sociological Factor

**Published:** 1905-01

**Authors:** R. R. Kime

**Affiliations:** Atlanta, Ga.


ATLANTA

### Journal-Record of Medicine.

Successor to Atlanta Medical and Surgical Journal, Established 1855, and Southern Medical Record, Established 1870.

OWNED BY THE ATLANTA MEDICAL JOURNAL CO.

**Published Monthly.**

VOL. VI. JANUARY, 1905. No. 10.

BERNARD WOLFF, M.D., EDITOR, Nos. 319–20 Prudential.

M. B. HUTCHINS, M.D., BUSINESS MANAGER, 1007-1008 Century Bldg.

J. N. LeCONTE, M.D., ASSOCIATE EDITOR.

### ORIGINAL COMMUNICATIONS.

### TUBERCULOSIS AS A SOCIOLOGICAL FACTOR.

By R. R. KIME, M.D.,
ATLANTA, GA.

Tuberculosis affects all mankind. The rich and the poor, the high and the low, the white and the black, the young and the old, the learned and the unlearned, are directly or indirectly influenced by the "Great White Plague."

No nationality and no clime is entirely exempt from its devastating influence.

No disease is so universal nor more closely allied with the social and economic life of mankind.

It is not confined to mankind, but invades the lower order of animals in such a way as to menace and affect the welfare of man, especially from a physical and economical standpoint.

While tuberculosis is due in many instances to physical and moral sin, yet it is often visited upon the innocent and unsuspecting. It produces more disease, suffering and deaths than any other disease known to mankind, yet it is preventable and curable.

While heredity does not play as prominent a part in its production as once thought, yet it develops a suitable soil for the propagation of tubercular germs. Such persons have impaired vitality, weakened constitutions and are more susceptible to disease. In the battle of life they are unable often to withstand the hardships, privations and exposures incident to earning a living. The pecuniary loss is a social factor not to be forgotten in considering tuberculosis.

Pneumonia of late is becoming a formidable rival in death-rate, for it is an acute disease and runs its course rapidly, while tuberculosis is necessarily chronic. Besides the loss of life, the invalidism requiring nursing, loss of time, treatment, medicines and care greatly increase the expense that must be borne by the relatives, friends or the State.

Dr. George M. Gould (*Philadelphia—Amer. Med., Sept. 7, 1901*) states: "Of 70,000,000 people now living in the United States 10,000,000 will die of tuberculosis.

Osler says: "1,200,000 of our people have the disease at any one time. One third of the deaths occurring between the ages of fifteen and sixty are said to be due to it."

The same author states "about 9,000 die annually in New York City from pulmonary tuberculosis, about four times this number suffering from the disease, three fourths of whom are living in the tenement-house districts. The actual economic value of these deaths and of sickness is over $8,000,000 per annum."

"In the registration area for about 21,000,000 people tuberculosis has been responsible for nearly 12 per cent. of all deaths."

Dr. Harris states one-fifth of all the diseases in Union Springs in 1895 were due to tuberculosis, and all occurred among the blacks, "a larger death-rate in the blacks from tuberculosis alone than the total white death-rate."

The accompanying table shows the relative death-rate in the colored and white races, also the monetary loss to each city from tuberculosis.

Each life is valued at $1,000, while the care, board, nursing, medical attention and medicines are valued at $1,000 each, being the lowest estimate of any so far given.

It is of interest to note the average death-rate from tuberculosis in the white race for the three years given in the different cities.

The figures also establish the fact of a higher death-rate in the negro almost three times as great as in the white race.

These figures give data for consideration by the sociologist, economist and sanitarian.

It is recognized that the Indian is most susceptible to tuberculosis; the negro is next in susceptibility. The negro being more closely allied to us and more numerous, with a death-rate from tuberculosis more than double that in the white race, it becomes a very important social factor.

Before the war tuberculosis was almost unknown in the negro, now fifty to seventy-five per cent. have tuberculosis or some specific disease, one or both. They are unhygienic, many abhor water, and so live as to be hot-beds for the development and spread of contagion.

They serve as cooks, chambermaids and nurses in our homes, with but little knowledge and care as to the spread of contagion. They nurse your children and convey to them the germs of disease, wash your clothing in unsanitary places, and are often walking volcanoes erupting tubercular germs that do their deadly work without regard to color or social equality. Sociologically, the race question means far more than social equality.

The Jewish race has been recognized as somewhat immune to tuberculosis. As their circumstances and environment change they seem to become more susceptible to the disease. Dr. Theodore B. Sachs (*A. M. A. Jour.*, Aug. 6, 1904) states in Chicago the death-rate is 1.51 per 1,000 of Jewish population. After an extensive investigation and study of the subject he concludes: "The so-called immunity of the Jews from tuberculosis is greatly overestimated."

Lillian Brandt finds that cities of 50,000 to 100,000 have a higher death-rate from tuberculosis than other cities. He also finds, that of twelve largest cities in the United States, New Orleans has the largest death-rate from tuberculosis—3.18 per 1,000; that San Francisco is next with 2.64, with Buffalo lowest, being 1.29. "Of the States, California leads with the highest death-rate from tuberculosis; then follows Tennessee, Kentucky, South Dakota, West Virginia," etc.

Dr. Lawrence F. Flick (director of Phipps Institute, Philadelphia) makes the following propositions:

"1. Tuberculosis is a house disease. It runs its course and proves fatal in the house; without the house it could not continue to exist.

"2. Tuberculosis is a family disease.

"3. Tuberculosis is in a measure an occupation disease.

"4. Tuberculosis is a disease of the poor, down-trodden and debased. The tubercle bacillus does not grow well in healthy tissue. Overwork, underfeeding, underairing, dissipation, physical injury to tissues by traumatism or other diseases, are usually the forerunner of tuberculosis.

"5. Tuberculosis is a slow, long-drawn-out disease, which as a rule kills by inches. Even those cases known as galloping consumption have had a long period of unrecognized quiescence. The average duration of tuberculosis probably is ten years, most of the time being a period of delicate health merely.

"6. Tuberculosis is a curable disease.

"7. Tuberculosis can be prevented. That which is due to life is perpetuated by reproduction; stop the reproduction and it becomes extinct. The tubercle bacillus only can reproduce itself in a host. Its life outside of a host is dormant. . . . It is through pus or sputum, and this alone, that tuberculosis can be conveyed from one person to another. Science has demonstrated that tuberculous matter thrown off can be sterilized or destroyed, and, if done at the moment it is thrown off, absolutely prevents new implantations. Practically the prevention of tuberculosis, then, is a question of ways and means how to bring every tuberculous person under control.

"8. Tuberculosis exists in every part of the world, in every climate and all seasons of the year. It has existed at all times of which we have any historic record. It is the most prevalent of all diseases, and causes more deaths than any other disease. It causes one-seventh to one-tenth of all the deaths throughout the world. The treatment and control of tuberculosis is, therefore a universal problem. It must be worked out in every part of the world, and the methods used must be such as can be used in any part of the world.

"9. The extermination of tuberculosis in animals must go hand in hand with the extermination of tuberculosis in human beings. That animal tuberculosis can be conveyed to human beings is a settled fact."

Dr. Flick also states: "Tuberculosis can be successfully treated anywhere; climate has practically nothing to do with the matter. Modern scientific treatment of tuberculosis means a carefully selected diet, life in the open air, regulation of exercise, and such medication as will help injured organs to do their work in a physiologic way."

Statistics demonstrate that certain occupations, crowding in illy ventilated apartments, residence in tenement-house sections of cities, all tend to increased development of tuberculosis.

Tuberculosis has become so interwoven into our social fabric that it is a factor of great importance in the future development of mankind. No community, city, State, race or nationality is free from its blighting influence.

To control and prevent tuberculosis will require not only a study of the disease itself but a study of our social and economic conditions. It will also require the combined efforts of the medical profession, boards of health and municipal governments, local, State and national. The combined efforts of all directed against the disease, in the human race and animals, is essential to success. It will require education, money, legislation and moral support for a successful crusade against "the great white plague."

Education should commence in our schools. Education not alone as to dissemination of contagious diseases, but as to those vices that tend to produce disease. It is short-sighted economy to spend all our energies combating a disease and at the same time go on assiduously preparing the soil for its development. Our system of education is faulty in developing children of impaired vitality and lessened power of resistance to disease. Our moral standards admit vice, dissipation, intemperance, tenement-house abuse, child labor, sweat-shops, etc., that tend to produce degeneration and develop disease. While these things do not develop the tubercular germs *de novo*, they prepare the human race for the invasion and development of the same.

The universal use of patent medicines and advertised "sure-cures" for consumption is a great evil and does much to increase the death-rate from tuberculosis. Such advertisements in secular and religious papers should be prohibited. Intemperance, or alcoholism, is a powerful factor in the production of tuberculosis. It devitalizes and degenerates the race, it produces neuroses, disease of stomach and other vital organs, and prevents proper nutrition. The children of alcoholics are neurotics, of lowered vitality, and are more susceptible to other diseases as well as tuberculosis. Any remedy that produces the degeneration and diseases of the vital organs of the body that alcohol does certainly is not entitled to be classed as a "specific for consumption."

The medical profession is to a great extent responsible for the popular idea and use of alcholics for consumption.

Such a conception has wrought much harm, and the profession should use its influence to combat such universal use. On this subject we make a few quotations.

Dr. Lawrence E. Flick, Philadelphia: "Opiates and alcohol should never be used" . . . in tuberculosis. "With the older physicians, the best that can be hoped for is to convince them that tuberculosis is contagious, curable and preventable, and to induce them to drop old methods of treatment with alcohol, opiates and depressants, substituting therefor common-sense diet with outdoor life."

Dr. S. A. Kopf, New York, states: "There is another point in regard to alcohol and tuberculosis I wish to emphasize, and that is the idea that alcohol is a remedy or even a specific for consumption. There has never been a greater mistake made. Alcohol has never cured and never will cure tuberculosis. It will even prevent or retard recovery. It is like a two-edged weapon—on one side it poisons the system and on the other side it ruins the stomach and thus prevents this organ from properly digesting the necessary food."

Bungé (Physiologic and Pathologic Chem.): "It is evident to every reasonable being that alcoholic drinks can only be prescribed for acute diseases, and should never be allowed in chronic disorders."

The family physician, revered and time-honored in the past, overshadowed by the specialist in the present, will be the important factor in the future in the control of tuberculosis. The all-round well-qualified general practitioner is the backbone of the profession, and should be held in high esteem and close relation to every family as their confidential adviser and counselor. He it is to whom seventy-five per cent. of the cases of incipient tuberculosis will first go for advice and treatment, at a time when they are curable and not a walking menace to the community in which they live. This is the time at which the greatest good can be accomplished, not only in the cure but in the prevention of the spread of the disease by timely counsel and advice. This is not only within the province but the duty of the "family doctor." The medical profession should use its influence against the present tendency to the mushroom development of pseudo-specialists, and urge a higher standard of excellence of the general practitioner in the diagnosis, prevention and treatment of disease. For a successful crusade against tuberculosis, as well as other diseases, such qualifications are essential. Through the general practitioner the public must be principally educated, protected and treated.

Sanatoria for tuberculosis will accomplish great good, but they can only reach a small part of the vast number affected. Each State and municipality should support such an institution.

1st. As an educational factor for such as come under its influence, directly or indirectly.

2d. For those unable to secure proper treatment, care and diet, or for those of moderate means that need the aid of such an nstitution.

3d. As a means of preventing the spread of contagion.

Sanatoria can not accomplish all that is needed. Dispensaries for the treatment of tuberculosis in every city and town, under the direction of competent physicians, can accomplish equally as much if not more good. Such dispensaries, acting in conjunction with the boards of health, can accomplish a vast amount of good in the prevention and relief of this disease. However much sanatoria and dispensaries may accomplish, we place over and above each the general practitioner in opportunities and responsibilities. These are all educational factors and education is the key-note to the prevention of this disease.

Each State should have a plain, simple, concise, practical text book, not only on tuberculosis but other common contagious and infectious diseases, for use in our public schools, so that children may be taught the elementary principles in the prevention and control of such diseases.

All cases of tuberculosis, in country as well as in cities, should be reported to the boards of health, so that when patient dies or moves the room can be properly disinfected. The boards of health should furnish each family, when case is reported, printed slips giving the manner of propagation of the disease, the manner in which the contagion is spread, giving the best recognized methods of prevention and disinfection.

In conclusion I would state, from present indications, tuberculosis is slightly on the decrease.

By the combined influence of the medical profession educating the general public, with the cooperation of the boards of health, the influence of the various tuberculosis commissions, the establishment of dispensaries and sanatoria, we may hope to accomplish much good in the near future.

Every profession, every municipality, every nationality, is interested in the prevention and control of tuberculosis. Funds, education and legislation are needed for success. Tenement-house districts must be renovated, crowding in factories, workshops, etc., must be avoided, dissipation and intemperance must be lessened, boards of health must be supplied with funds to execute laws and control contagion, including bovine tuberculosis as well as human. Boards of education must see that schoolchildren have proper instruction along this line, the general public must be educated by lectures and distribution of literature, and in this section the negro must receive special care and attention along these lines.

